# Morbid Anatomy

**Published:** 1833-07-01

**Authors:** 


### WORKS ON MORBID ANATOMY.

I. Anatomie Pathologique du Corps Humain, &c. Par *J. Cruveilhier*, Professeur d'Anatomie à la Faculté de Medecine de Paris, &c. &c. Livraisons quinzieme et seizieme. Bailliere, Paris et Londres.

II. Illustrations of the Elementary Forms of Disease. By *Robert Carswell*, M.D., Professor of Pathological Anatomy in the University of London, &c. &c. Fasciculus second. Carcinoma. Price 12*s*.

We have on former occasions observed that the plan of M. Cruveilhier's work is different from that adopted by Dr. Baillie, in his System of Morbid Anatomy. Dr. Baillie's is the method adopted by two contemporary illustrators of the same subject, Drs. Carswell and Hope. All these gentlemen have given general descriptions of lesions, without allusion to particular cases. Dr. Hope, indeed, does refer to some, but not to such an extent as to invalidate the general plan. M. Cruveilhier's fasciculi, on the contrary, are made up of references to cases, and the drawings of morbid appearances are taken from the subjects of the cases related in the text. There is this disadvantage attendant on Cruveilhier's mode of proceeding—the work must necessarily be extremely diffuse. It will, however, be very valuable for reference, and will furnish instructive matter in detail.

The drawings are very inferior to those of our own countrymen, and as we always give a fair portion of praise to our foreign brethren, we do not think that we shall be accused of national partialities in making this remark.

The sketching is stiff, formal, and in many instances caricatured, and the colouring highly exaggerated. Cannot M. Cruveilhier amend this?

The fifteenth fasciculus is chiefly devoted to diseases of the fœtus. It comprises apoplexy—diseases of the lungs and thymus of the fœtus—diseases of the pharynx—hydrocephalus—and a case of absence of the cerebellum. There is also a case of hernia through the thyroid foramen. The sixteenth fasciculus treats of diseases of the placenta—heart—liver—spinal marrow (spina bifida)—uterus—and veins. We will take the fifteenth fasciculus first.

### On the Apoplexy of Newly-born Children.

It appears from the researches of M. Cruveilhier at the Maternité, on the cause of death of still-born children, that apoplexy is that cause in a third of those who die during the progress of labour. He has observed this in almost all the cases, usually considered as asphyxia or congenital weakness.

The constant anatomical character of the apoplexy of newly-born children is, an effusion of liquid blood within the cavity of the arachnoid. Most frequently the effusion is limited to the surface of the cerebellum, sometimes it covers at the same time the posterior lobes of the cerebrum, and in some instances the cerebrum and cerebellum are covered with a layer of blood, the source of which it is sometimns difficult to discover. The extravasation rarely occupies the cavity of the ventricles, but M. Cruveilhier has seen three such cases. He has never witnessed extravasation into the substance either of the cerebrum or cerebellum, although it is occasionally intensely injected. In all the cases which he has seen the spinal dura mater was distended with liquid blood, contained both in the cavity of the arachnoid and in the subarachnoid cellular tissue.

These effusions are often accompanied with collections of blood under the hairy scalp, and ecchymoses and small collections are frequently met with between the peritoneum and the cranial bones.* Once M. Cruveilhier saw coagulum effused along the whole length of the superior longitudinal sinus, between the dura mater and the bone; and on another occasion the pericranium of the two parietal and occipital bones was separated from them by a thick layer of coagulum. Our author observes that this separation may account for those occipital abscesses that we see occur during the first days of life, and in which the bones are laid bare. He attended a child in whom the large part of the occipital bone came away in three fragments; the child lived.

All apoplectic children are not born dead. In a tolerable proportion respiration is established, either spontaneously or in consequence of the exertions of the practitioner, and they live 24 or 48 hours, or even three or

---

* In one of the periodical journals a case of this sort has recently been published, and a good deal of surprise expressed at the occurrence. We have M. Cruveilhier's authority for considering it frequent. We ourselves saw a case, in which the collection formed a large fluctuating tumor on the scalp. We had no difficulty in deciding on its nature, but an eminent surgeon had never witnessed a similar case.—Ed.

four days in a state of weakness, torpor, immobility, and diminished temperature. Probably some survive when the extravasation is not very great. M. Cruveilhier has never observed paralysis.

The cause of this fœtal apoplexy is in many cases inscrutable. So far from its being due to the application of the forceps, M. Cruveilhier believes that this frequently prevents it. In the majority of instances it is probably owing to the length of the labour. But our author has seen it after an ordinary birth, and even after a very rapid delivery, whilst, on the other hand, labour has lasted forty-eight hours or longer without any accident happening to the child. M. Cruveilhier seems to suppose that constriction of the neck by the cord, or by the neck of the uterus and the external soft parts after the issue of the head, or after that of the trunk in cases of turning or feet-presentation, may produce the disease. Compression of the umbilical cord in a case of presentation of the cord, has been a very manifest cause. M. Cruveilhier also supposes that, in many cases, after the escape of the waters, the uterine contractions on the cord may be an efficient agent.

M. Cruveilhier looks on the affection as always produced by mechanical means, and thinks that a speedy termination of the labour would prevent it in the greater number of cases. He has seen retropulsion of the cord, when this presented, practised successfully at the Maternité; and in feet-presentations a speedy application of the forceps, after the exit of the trunk, will save many children.

The cerebral apoplexy is sometimes accompanied with extravasations in the lung, in the thymus, whilst the liver and spleen are gorged with blood, and the gastro-intestinal mucous membrane is much injected. Our author relates some cases to some of which we will allude.

*Case* 1. A child was born dead in the first position of the vertex. The labour was natural and lasted sixteen hours. On opening the head, a great quantity of black blood was found around the cerebrum and cerebellum, with more liquid blood in the spinal canal. There was no perceptible compression of the cord.

*Case* 2. *(Third.)* A child presented in the third position of the right shoulder, that in which the head answers to the left iliac fossa; the waters had long escaped. An attempt at turning by an ill-informed person having failed, it was necessary to repeat it. When the trunk was extracted the head remained for a long time, the neck of the uterus being contracted on that of the child who was born dead, although active motions had been felt a few minutes previously. On examination, there was considerable injection of the hairy scalp, periosteum, and bones; the spinal dura mater was very distended and blueish, and the spinal marrow immersed in liquid blood. A thin layer of blood surrounded the cerebrum, a thicker one the cerebellum.

Five other cases are related by M. Cruveilhier. In one the child was born dead, with a vertex presentation; there was suspension of the pain after the issue of the head, and the birth of the trunk was slow. In the next the child was born dead, with a head-presentation. In the next the child died an hour after birth. In the next the child died after the issue of the cord. And in the last the child was hydrocephalic, and born dead after a natural labour.

### DISEASES OF THE LUNG, THYMUS, AND PANCREAS IN THE FŒTUS.

M. Cruveilhier observes that during gestation the ovum may be considered a part of the mother, and is liable to disease as she is. Thus it may be affected with pleurisy, pneumonia, peritonitis, chronic inflammation of the intestines, ascites, hydrothorax, syphilis, eruptions, even scirrhus. M. Cruveilhier appends cases of many of these affections, and as we fear that practitioners in this country are not sufficiently aware of the disorganizations that occur in the fœtus, we shall select the most striking for notice.

CASE 1.—*Double Pleurisy—death thirty-six hours after birth.* A woman had experienced for eleven days before her confinement a sensation of oppression amounting even to suffocation, which required bleeding and sinapisms. She was relieved, but fever continued till the moment of accouchement, which was natural and at the proper time. The child was well-formed but small and weak; it languished for thirty-six hours, when it died. It was marked as dying of "congenital weakness." On examination there was pleurisy on each side, with effusion of a turbid liquid and false membrane; the base of the lungs was indurated, or rather in the condition of the lung of children who have not respired.

CASE 2.—*Peritonitis and Pneumonia in a Child, dead three hours after birth.* On dissection of this child, which was born naturally and with a vertex presentation in the first position, a great quantity of yellowish serum, with flakes of lymph, was found in the cavity of the peritoneum. The internal surface of the stomach presented a punctuated and linear redness. The lymphatic glands were large and indurated. The lungs presented externally a speckled appearance, owing to numerous red irregular indurated masses dispersed through the organ; they might be termed large tubercles.

M. Cruveilhier remarks that the appearance of the mucous membrane of the stomach alluded to in this case is very common in the fœtus.

CASE 3.—*Spinal Arachnitis—death on the fifth day.* A child, that appeared rather prematurely born, took the breast for three days, but became motionless and cold on the fourth. M. Cruveilhier saw it in this state, and thinking it debility, prescribed an aromatic bath, which usually succeeds in cases of that description. On the next day the child died. On examination there was spinal arachnitis; the sub-arachnoid cellular tissue of the medulla was infiltrated with pus, which extended around the pons, and along the fissures of the cerebrum.

M. Cruveilhier supposes that the arachnitis was subsequent to birth.

CASE 4.—*Dropsy and Purpura in a seven months' Fœtus—death twelve hours after birth.* This child was anasarcous, and the spots of purpura covered the whole surface of the body and mucous membrane of the tongue. On examination there was serous fluid in the abdomen, the liver was reduced to half its accustomed dimensions, and there were some purple spots on the mucous membrane of the intestines. There was hydrothorax on the right side. Nearly all the muscles presented extravasations of blood in their substance, as did the pericranium and mucous membrane of the pharynx. The cerebral sub-arachnoid cellular tissue was infiltrated with serum, the thoracic duct distended by bloody serous fluid.

Case 5.—*Dropsy and Purpura—death one hour after birth.* In this child there was a great quantity of serum in the pericardium, a little in the pleuræ, some in the abdomen. The liver was of greenish colour and very dense. There were ecchymoses beneath the scalp, in the muscles, &c.

The mother of this child was in a state of cachexia, the result of syphilis and mercury. She was delivered at the full time, and the labour was easy. The mother of the infant in the preceding case had been for a long time in a weakly condition.

Case 6.—*Chronic Inflammation of the Thymus and Pancreas, &c.* The child was born at the full time, and the vertex presented. The feet were stripped of epidermis and livid. The child died after breathing for a few minutes. On examination a collection of pus was found beneath the sternum; it occupied the thymus, which was of large dimensions, and divided into cellulæ, those above containing tuberculous matter, those below filled with pus.* The lungs were large, heavy, and only permeable in a few points, their tissue being dense and of a rose colour. The liver was extremely small. The pancreas was of lardaceous appearance, and resembled in structure a scirrhous breast. It adhered to the right renal capsule and right kidney.

M. Cruveilhier observes that, in some cases, there appears to be retention and collection of the liquor of the thymus. In a child that died of marasmus at the age of six months, the thymus was found of double its natural size, and full of tubercles and purulent collections; the lungs were perfectly sound.

Case 7.—*Induration of the Pancreas and Lungs, &c.* A child died immediately after birth, the pancreas was indurated and resembled scirrhus, and the lungs were almost entirely in a state of induration. The intestines presented a curious condition. There were whitish, elliptical patches, with considerable thickening of their parietes; the patches were strewed with small ulcerations, and in some points the thickening was such that the cavity of the intestine appeared completely obliterated.

Case 9.—*Obliteration of the Orifice of the Pulmonary Artery, and Aneurism of the Right Side of the Heart in a Fœtus.* The child was born at eight months and a half in a state of extreme weakness; the respiration was imperfect, difficult, and almost convulsive; it died on the fifth day. On examination the heart was of enormous size, filling more than half the thorax, and pressing back the lungs, which were diminutive. The right chambers formed seven-eighths of the organ, the left were so small as to merely resemble an appendix. The tricuspid valve was fixed to the ventricular parietes, and vegetations occupied the free edge of the valve. The

---

* Sir Astley Cooper, in his work on the Thymus Gland, observes that, in an experience of forty years, he has seen but one case of disease of the thymus. But Sir Astley is not speaking of the fœtus, and besides the worthy Baronet has not practised midwifery.

orifice of the pulmonary artery was completely obliterated, but otherwise the vessel and its ramifications were sound. The fossa ovalis was large, the foramen was a mere chink, in which a coagulum was engaged.

### DISEASES OF THE LUNGS IN THE FŒTUS.

Diseases affecting the tissue of the lung are incontestibly the most frequent to which newly-born children are subject. In many cases the disease appears to have existed for a few days only before birth, or even to have been the consequence of the commencement of the act of respiration. The partial development of the lungs, or pulmonary infiltration observed in the fœtus born before its time, and which breathes for several hours or even days, are evidently, in M. Cruveilhier's opinion, the result of the establishment of the function of respiration in lungs not yet adapted to it.

In the greater number of cases the disease has existed for several days or even months. The lungs then present different appearances. Sometimes they display the lobular pneumonia. Lobules, or groups of lobules, indurated or infiltrated, are dispersed in the midst of healthy tissue. Sometimes the lungs are extensively affected; M. Cruveilhier has seen both invaded throughout at once. The altered tissue sometimes resembles that of the pneumonia of the newly-born, at others it is completely carnified, granular, and the lobules represent glandular grains perfectly distinct. Lesion of the lungs is so frequent in the fœtus, that M. Cruveilhier has no hesitation in asserting that, *as many newly-born children die of disease of the lungs as adults*.

Such children die with asphyxia, but this differs from the asphyxia which is said by accoucheurs to be the cause of death, inasmuch as the latter is the mere result of the labour. The greater number of those who are said to die of such asphyxia do in reality die of apoplexy. The newly-born may live with a much less sound quantity of lung than the adult, and for reasons sufficiently obvious. Induration of the lungs may exist in the fœtus with embonpoint, and the appearance of perfect health. The bronchi are frequently filled with thick mucus, as in acute catarrh. The lungs of the fœtus may display much more extensive lesions than are sufficient to destroy the life of the adult.

M. Cruveilhier relates a considerable number of cases illustrative of the lesions alluded to. We will take only two or three.

CASE 1. (3.)—*Lungs nearly entirely infiltrated with Blood.* The infant was born very weakly, and so continued for twenty-four hours, when it died. There was considerable effusion of bloody serum in the pleuræ. The lungs were indurated and infiltrated with blood, which flowed as from a sponge; one only presented some permeable lobules.

CASE 2. (7.)—*Phlyctenæ—some Pulmonary Lobules impermeable—Pulmonary Catarrh.* The phlyctenæ were situated on the feet, hands, and left upper eyelid. It lived thirty-six hours. Some lobules of the lung were impermeable from sero-sanguinolent infiltration, and one was infiltrated with pure blood. The trachea, the bronchi, and their divisions were filled with thick mucus.

A great number of children affected with syphilitic pustules have died of pneumonia or other lesion of the lungs, originating anterior to birth. Four cases are related. In the first there were cutaneous pustules, and the lungs were infiltrated here and there with blood and serum. In the second case the cutaneous pustules were connected with masses of induration in the lung, containing pus, and suppuration between the dura mater and frontal bone. In the third case the cutaneous pustules were accompanied with a greyish induration of the lungs throughout their whole extent; the spleen was much enlarged. In the fourth case the cutaneous pustules were accompanied with complete induration of both lungs.

### Diseases of the Mouth and Pharynx.

Figures 1, 2, 3, of the third plate of this fasciculus are devoted to the delineation of aphthæ, le Muguet. M. Cruveilhier observes, that this affection is sometimes confined to the buccal membrane, sometimes extends to the mouth and pharynx, and sometimes attacks the œsophagus into which it penetrates more or less deeply. It is most commonly abruptly stopped at the termination of the epidermis of the œsophagus at the cardia. Under the false membranes that constitute the affection, and which are sometimes confluent, the mucous membrane is invested with its epidermis, and presents no other alteration than a redness, slight when the patches are distinct, intense when they are confluent. The muguet rarely penetrates into the air-tubes. Sometimes the circumference of the superior orifice of the larynx is studded with it, whilst the mucous membrane within is free. But M. Cruveilhier delineates one instance of implication of the ventricles of the larynx. The linear character of the pseudo-membranes of the œsophagus depends on the longitudinal folds of the mucous membrane.

M. Cruveilhier remarks that the muguet is nothing else than pseudo-membranous inflammation of the mucous membrane of the mouth. All ages are subject to it, though in the adult it is rarely idiopathic, but almost always symptomatic of some grave lesion of the intestinal canal, and appearing at a more or less advanced period of acute and chronic diseases, sometimes even during a false convalescence. It usually bodes a fatal termination, especially where it resists treatment. It may extend in the adult, as in the infant, to the pharynx and œsophagus, which our author has on several occasions found filled with a pultaceous whitish or brownish matter. In newly-born infants it is often epidemic, and it is endemic in certain hospitals where the wards are cold, humid, and ill-ventilated. This is the case with the hospital of Limoges, when the disease formerly committed great ravages. M. Cruveilhier doubts the advantage of the hospital system for the newly-born. The milk of a good nurse and pure air are much more advantageous than medicine.

### Follicular Ulceration of the Stomach.

M. Cruveilhier remarks that this form of ulceration has been described by M. Belliard, who has published fifteen cases. In eight of these the children were from four to six days old; six were from eight to twelve; one was aged three weeks; proving, so far as a few facts can do so, that the nearer birth, the greater the prevalence of this affection. Several of the patients had severe lesions in other organs; one only, aged four days, had no other lesion than ulceration of the stomach.

Figures 4, 5, 6, of plate III. represent this lesion in the stomachs of children who died, one on the eighth day, another on the fifteenth, and the third one month after birth. In all, the ulcers were more or less numerous, and some had run into each other.

The remainder of the fifteenth fasciculus is occupied with some considerations on, and cases of the removal of, the cerebral matter in hydrocephalus; a case of complete absence of the cerebellum, observed in a girl who died in her eleventh year, and a case of hernia through the foramen ovale. Further reference to these subjects will be found in the foreign department of the Periscope of this number, and we fear we must waive all consideration of the sixteenth fasciculus until our next. Before we quit M. Cruveilhier, we must take the liberty of recommending the present notice of the organic lesion of infants to the attention of our brethren, too many of whom are ignorant of the morbid anatomy of the fœtus. Indeed it is probable that few are aware of the frequency of its structural changes.

We can give but brief salutation to Dr. Carswell, whose work is the second on our list. It made its appearance too late to permit us to notice it at length, but a further reference to it also will be found in our Periscope.

It is devoted to the elucidation of carcinoma, and contains four plates, of which it is but justice to observe that they are very beautifully executed. Plate I. contains three drawings of carcinomatous growths of the stomach. The external characters of the disease, its section, and the submucous tissue from which it chiefly springs are well delineated. Plate II. contains also three drawings; they are intended to portray the medullary form of carcinoma, and the transition stages between it and scirrhus. Plate III. represents carcinoma in the stomach, duodenum, rectum, and female bladder. It consists of five figures. The first shews the affection of the rectum. The disease seems essentially seated in the submucous tissue, but the cellular of the muscular texture is also altered. The second displays a cancerous ulcer of the stomach near its cardia, also essentially seated in the submucous tunic. The third shews medullary tumour of the duodenum, confined to its mucous membrane; the lacteals are filled with cerebriform matter, and the lymphatic glands contaminated. The last figure represents what is called cauliflower excrescence of the female bladder; the mucous and the submucous tissue is affected. Plate IV. represents carcinoma in the liver, and does so very well. The development of the morbid growth from a small deposite up to a large one, or the confluence of several, with central softening and irregular vascularization is displayed. But the most interesting part of this plate is the delineation of the cerebriform matter in the veins, passing thus from the morbid mass to the circulation. Thus we find this morbid degeneration in both veins and absorbents, and we need not wonder at the almost universal contamination of system so unfortunately witnessed in this disease.

We need not pronounce any formal eulogy of the plates; they are, as we have said, remarkably well executed. We would suggest to Dr. Carswell to outline as definitely and yet as little formally, and to colour as truly as possible. The work is one which should be encouraged by all medical lovers of art as well as of science, and indeed no practitioner who aspires to respectability should at this time of day neglect to purchase one or other system of plates of morbid anatomy.

We again refer to our Periscope for a critical notice of the letter-press.

